# Contaminated dicloxacillin capsules as the source of an NDM-5/OXA-48-producing *Enterobacter hormaechei* ST79 outbreak, Denmark and Iceland, 2022 and 2023

**DOI:** 10.2807/1560-7917.ES.2023.28.9.2300108

**Published:** 2023-03-02

**Authors:** Charlotte Nielsen Agergaard, Lone Jannok Porsbo, Thomas Vognbjerg Sydenham, Sanne Grønvall Kjær Hansen, Kat Steinke, Sanne Løkkegaard Larsen, Kristján Orri Helgason, Frank Hansen, Kasper Thystrup Karstensen, Anna E Henius, Barbara Juliane Holzknecht, Lillian Søes, Kristian Schønning, Mikala Wang, Nina Ank, Anna Margrét Halldórsdóttir, Ólafur Guðlaugsson, Anette M Hammerum, Anne Kjerulf, Brian Kristensen, Henrik Hasman, Ulrik Stenz Justesen

**Affiliations:** 1 Department of Clinical Microbiology, Odense University Hospital, Odense, Denmark; 2 Infectious Disease Epidemiology and Prevention, Statens Serum Institut, Copenhagen, Denmark; 3 Department of Clinical Microbiology, Landspitali University Hospital of Iceland, Reykjavik, Iceland; 4 Department of Bacteria, Parasites and Fungi, Statens Serum Institut, Copenhagen, Denmark; 5 Department of Clinical Microbiology, Copenhagen University Hospital - Herlev and Gentofte, Herlev, Denmark; 6 Department of Clinical Microbiology, Copenhagen University Hospital – Amager and Hvidovre, Hvidovre, Denmark; 7 Department of Clinical Microbiology, Copenhagen University Hospital - Rigshospitalet, Copenhagen, Denmark; 8 Department of Clinical Microbiology, Aarhus University Hospital, Aarhus, Denmark; 9 Department of Clinical Microbiology, Aalborg University Hospital, Aalborg, Denmark; 10 Centre for Health Security and Communicable Disease Control, Directorate of Health, Reykjavik, Iceland; 11 Department of Internal Medicine, division of Infectious Diseases, Landspitali University Hospital of Iceland, Reykjavik, Iceland

**Keywords:** Outbreak, Enterobacter hormaechei, CPO, dicloxacillin, antibiotic

## Abstract

From October 2022 through January 2023, nine patients with NDM-5/OXA-48-carbapenemase-producing *Enterobacter hormaechei* ST79 were detected in Denmark and subsequently one patient in Iceland. There were no nosocomial links between patients, but they had all been treated with dicloxacillin capsules. An NDM-5/OXA-48-carbapenemase-producing *E. hormaechei* ST79, identical to patient isolates, was cultured from the surface of dicloxacillin capsules in Denmark, strongly implicating them as the source of the outbreak. Special attention is required to detect the outbreak strain in the microbiology laboratory.

*Enterobacter hormaechei* can cause various infections, such as bacteraemia, urinary tract and intra-abdominal infections and is a common cause of healthcare related infections [1-3]. Furthermore, NDM-5-producing *E. hormaechei* has been detected in a pork production chain [4]. Within 1 week in January 2023, we identified with *E. hormaechei* ST79 carrying *bla*_NDM-5 _and *bla*_OXA‑48 _in the urine of three patients at the same Danish hospital department of clinical microbiology (DCM), Odense University Hospital (OUH). Descriptive epidemiological investigations at Statens Serum Institut (SSI) in Copenhagen showed no nosocomial link between the patients with the outbreak strain, indicating a community-associated source such as travel abroad or an ingested product. The DCM initiated interviews with the three patients to determine if a common source could be found.

## Patient interviews and potential source of the outbreak strain

The structured phone interview included questions on the medical history, travel abroad and within Denmark, visits to restaurants and grocery stores and specific questions on intake of meat, vegetables and nutritional supplements. One patient reported treatment with dicloxacillin capsules in the days before the outbreak strain was detected in the patient’s urine sample. Treatment with dicloxacillin capsules before the detection of the outbreak strain was also reported by the two other patients. The national pharmacy database confirmed that all three patients had been prescribed dicloxacillin 500 mg capsules from the same manufacturer.

Two of these patients had dicloxacillin capsules remaining (a 50-capsule and a 30-capsule box), which we collected on the day after the interview. Blister packs with 500 mg capsules (gelatine) of dicloxacillin from the 50-capsule and the 30-capsule box were wiped with 70% of alcohol and left to air dry. The blister packs were broken without touching the foil covering the capsules and each capsule was placed on 5% horse blood agar (SSI Diagnostica, Hillerød, Denmark). The capsules were rolled over the surface of the agar with heat-sterilised tweezers (cooled before handling the capsules). The capsules were then removed from the agar surface and pulled apart with tweezers and the content dispersed on 5% horse blood agar. The rest of the broken capsules were placed in 7 mL of serum broth of which 100 µL was cultured on 5% horse blood agar the following day. This procedure was done with two capsules from two different blister packs. All procedures were performed in a laminar airflow cabinet. Plates were incubated at 35 °C in ambient air.

After 16 h of incubation, growth of a solitary colony was observed from the capsule surface culture of the 50-capsule box but not the 30-capsule box. Phenotypic characteristics were identical to the strains previously detected in the three patients from OUH. European Committee on Antimicrobial Susceptibility Testing (EUCAST) disk diffusion confirmed the resistance phenotype (Table) [5]. One more identical isolate was cultured on the following day from the 50-capsule box.

**Table:** Phenotypic characterisation of the first *Enterobacter hormaechei* isolated from dicloxacillin 500 mg capsules, Denmark, February 2023

Phenotypic test	Specific characteristics
Identification with Bruker MALDI-TOF MS	Different species within the *Enterobacter cloacae* complex (*E. cloacae*, *E. kobei*, *E. hormaechei* and *E. asburiae*) with scores ≥ 2 (*E. bugandensis* was also suggested for two of the three patient isolates from Odense University Hospital)
Lateral flow assay (NG-test CARBA-5)	NDM-positive and OXA-positive
Antimicrobial susceptibility testing with EUCAST disk diffusion	Resistant: Ampicillin, amoxicillin-clavulanic acid, piperacillin-tazobactam, mecillinam, cefuroxime, ceftriaxone, cefepime and meropenem
	Susceptible: Ciprofloxacin, gentamicin, trimethoprim, sulphonamide and nitrofurantoin

EUCAST: European Committee on Antimicrobial Susceptibility Testing; MALDI-TOF MS: matrix-assisted laser desorption ionization-time of flight mass spectrometry; NDM: New Delhi metallo-β-lactamase; NG: next generation; OXA: oxacillinase.

## *Enterobacter hormaechei* ST79 carrying *bla*_NDM-5_ detections since 2020

**Figure 1:**
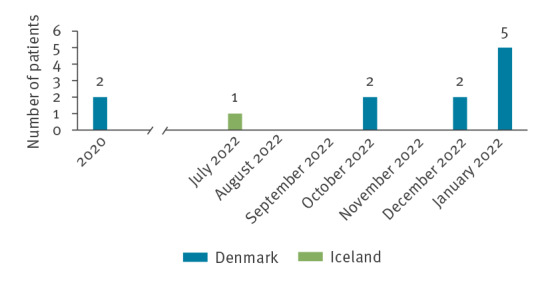
Number of patients with carbapenemase-producing *Enterobacter hormaechei* ST79, by month, Denmark and Iceland, 2020–January 2023 (n = 12)

Since 1 January 2014, all DCM have submitted carbapenemase-producing organisms (based on meropenem non-susceptibility) to the Reference Laboratory of Antimicrobial Resistance at SSI for confirmation and characterisation through whole genome sequencing (WGS) and analysis. The reference laboratory identified a total 11 patients with *E. hormaechei* ST79 and *bla*NDM_5_ in their database. Two cases were from 2020, while the nine most recent cases, including the three cases from OUH, occurred between October 2022 and January 2023 and originated from patients in four of five regions of Denmark (Figure 1). 

## Public health measures and international alert

Following laboratory confirmation of the strain phenotype cultured from the dicloxacillin capsules, an epidemiological investigation at SSI revealed that also the eight patients from other parts of Denmark, i.e. all 11 patients including the three from OUH, had received dicloxacillin 500 mg capsules from the same manufacturer before the detection of the carbapenemase-producing *E. hormaechei* outbreak strain. Based on this information, the Danish Medicines Agency recalled dicloxacillin 500 mg capsules manufactured by the specific company from pharmacies, including hospital pharmacies in Denmark [6]. They also notified the Rapid Alert Network, including medicines agencies within the European Union, United Kingdom (UK), Switzerland, United States, Japan, Australia, the United Nations Children’s Fund, and UNICEF and the World Health Organization. Furthermore, SSI posted alerts in the European Early Warning and Response System (EWRS) and European Centre for Disease Prevention and Control's EpiPulse platform[7].

Following the rapid alert issued by the Danish Medicines Agency, another patient case with the outbreak isolate was identified in Iceland. This patient had received dicloxacillin 500 mg capsules in July 2022 from the same manufacturer (Figure 1). Draft genomic data were available as part of the Icelandic surveillance programme and was shared with SSI for genomic comparison.

## Molecular characterisation

**Figure 2:**
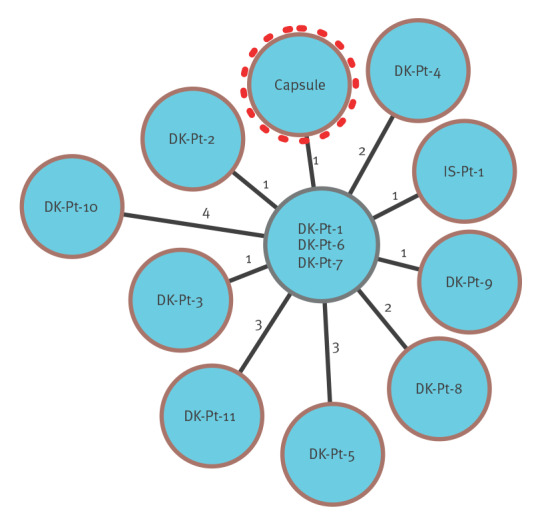
cgMLST analysis of *Enterobacter hormaechei* isolated from the dicloxacillin capsule and patient samples from Denmark and Iceland, 2020–January 2023 (n = 13)

The local DCM performed WGS with Nanopore (Oxford Nanopore Technologies, Oxford, UK) on the first capsule isolate, which confirmed it to be *E. hormaechei* ST79 (*E. cloacae* MLST scheme) with *bla*_NDM-5_ and *bla*_OXA-48_ located on two separate plasmids. Initially, we performed a single nucleotide polymorphism (SNP) analysis on rapidly generated Nanopore assemblies polished with routine Illumina data from the three most recent patients from OUH and the isolate from the dicloxacillin capsule. A close clonal relationship was established with an observed SNP distance of between 1 and 4. This finding was supported by cgMLST analysis (SeqSphere+ version 8.5.1, Ridom GmbH, Münster, Germany) comparing 3,441 core genes on a draft genome from the capsule isolate (generated by Illumina sequencing) together with the genome shared by the Icelandic reference laboratory. Subsequently, all 11 *E. hormaechei* ST79 carrying *bla*_NDM-5_ (nine isolates also carried *bla*_OXA‑48_) were analysed by core genome multilocus sequence typing (cgMLST) (SeqSphere+), with an allelic distance between 0 and 3. The dicloxacillin capsule isolate, the Icelandic patient isolate and all 11 Danish patient isolates were genotypically very closely related, with a maximum of 4 allele differences between the genomes (Figure 2). Examining all *E. cloacae* and *E. hormaechei* draft genomes (n = 2,699) present in the RefSeq database revealed one ST79, but with more than 200 allele difference in cgMLST analysis. 

cgMLST: core genome multilocus sequence typing; DK: Denmark; Pt: patient.

## Discussion

*Enterobacter hormaechei* can be part of the microbiota of the intestinal tract of humans and animals [8] and has caused outbreaks in animals as well as hospital settings [1,9]. It is a member of the *Enterobacter cloacae* complex, intrinsically resistant to many antibiotics [2] and can acquire resistance genes such as the carbapenemase genes *bla*_NDM-1_, *bla*_NDM-5_ and *bla*_NDM-7_ [3,4,10]. It can be difficult to identify as *E. hormaechei* because of the close relationship between the different members within the *E. cloacae* complex (*E. cloacae*, *E. asburiae*, *E.* *bugandensis, E. hormaechei*, *E. kobei*, *E. ludwigii* and *E. nimipressuralis*). 

We cultured NDM-5/OXA-48-carbapenemase-producing *E. hormaechei* ST79 from the surface of two 500 mg dicloxacillin capsules from a 50-capsule box from one patient and suspect that they were the source of an ongoing outbreak in Denmark with one additional confirmed case in Iceland. The outbreak strain may not be readily detected in the clinical microbiology laboratory for several reasons. Firstly, it will not necessarily be reported as *E. hormaechei* by laboratories using identification systems (e.g. MALDI-TOF MS) which do not discriminate well between members of the *E. cloacae* complex. Secondly, the three patient isolates from OUH were susceptible to most non-β-lactam antibiotics used to treat urinary tract infections (e.g. ciprofloxacin and trimethoprim) and may be overlooked unless meropenem is used for screening for carbapenem resistance. Thirdly, the plasmid carrying OXA-48 is not always present (only in 9 out of 11 isolates). 

This outbreak underlines the value of the Danish national surveillance system at SSI, supported by close collaboration with local DCM and the power of WGS to confirm outbreaks. The scale of the outbreak in Denmark has yet to be determined. It has been reported by the Danish Health Data Authority that ca 35,000 patients in Denmark have received dicloxacillin capsules from the same manufacturer in the period from September to December 2022. The European aspects of this outbreak are unknown, apart from the case in Iceland, which increases the timeframe and most likely the scale of the outbreak. Awareness and knowledge of this specific outbreak strain will help microbiology laboratories detect further potential cases. 

## Conclusion

There is an ongoing outbreak with an *E. hormaechei* ST79 carbapenemase-producing strain in Denmark linked to 500 mg dicloxacillin capsules from a single manufacturer, involving 11 patients since 2020. The Danish Medicines Agency recalled the product from pharmacies, including hospital pharmacies in Denmark, but it may still be available in private homes and practices. As the specific circumstances of the contamination are unknown, doctors are advised to be vigilant and investigate patients with unexpected findings of carbapenemase-producing *Enterobacter* complex species for exposure to dicloxacillin capsules. Clinical microbiology laboratories need to be aware of the difficulties in identifying the outbreak strain as it can be reported as different species within the *Enterobacter* complex, it is susceptible to most non-β-lactam antibiotics and it will not always carry or express the OXA-48 carbapenemase.
