# Author’s correction for Euro Surveill. 2023;28(9)

**DOI:** 10.2807/1560-7917.ES.2023.28.10.230309c

**Published:** 2023-03-09

**Authors:** Eurosurveillance editorial team

**Affiliations:** 1 European Centre for Disease Prevention and Control (ECDC), Stockholm, Sweden

In the article ‘Contaminated dicloxacillin capsules as the source of an NDM-5/OXA-48-producing *Enterobacter hormaechei* ST79 outbreak, Denmark and Iceland, 2022 and 2023’ by Agergaard et al., published on 2 March 2023, the following corrections were made on 3 March 2023 at the request of the authors: In Figure 1, the date for the Icelandic case was corrected from July 2022 to September 2022. This date was further clarified in the chapter on public health measures. In the Conclusion section, *Enterobacter* complex was corrected to *Enterobacter cloacae* complex.

